# Supplementary material for: Employing toxin-antitoxin genome markers for identification of Bifidobacterium and Lactobacillus strains in human metagenomes
Source: PeerJ. 2019 Mar 4;7:e6554. doi: 10.7717/peerj.6554 (PMC6404652; doi:10.7717/peerj.6554)
Supplement: Supplemental Information 1 — Strain diversity of Bifidobacterium in metagenomes. [file peerj-07-6554-s001.pdf]

Table S1 Strain diversity of *Bifidobacterium* in metagenomes

Groups	Representatives
**RM1**	
**I**	B. adolescentis ATCC 15703
**II**	B. bifidum ATCC 29521<br>B. bifidum JCM 1255
**III**	B. bifidum ATCC 29521<br>B. bifidum S17<br>B. bifidum JCM 1255
**IV**	B. bifidum PRL2010<br>B. bifidum ATCC 29521<br>B. bifidum BGN4<br>B. bifidum JCM 1255
**V**	B. longum subsp. longum KACC 91563
**VI**	B. bifidum BGN4
**VII**	B. adolescentis BBMN23
**VIII**	B. bifidum PRL2010<br>B. bifidum ATCC 29521<br>B. bifidum S17<br>B. bifidum BGN4<br>B. adolescentis 22L<br>B. bifidum JCM 1255
**IX**	B. breve JCM 7019
**RM2**	
**I**	B. longum subsp. infantis 157F<br>B. longum DJO10A
**II**	B. longum subsp. infantis 157F<br>B. longum DJO10A<br>B. longum NCC2705
**III**	B. animalis subsp. animalis ATCC 25527
**IV**	B. bifidum S17<br>B. bifidum JCM 1255<br>B. bifidum ATCC 29521<br>B. bifidum PRL2010
**V**	B. longum subsp. infantis 157F
**VI**	B. longum subsp. infantis 157F<br>B. longum DJO10A<br>B. longum NCC2705<br>B. longum 105-A<br>B. longum subsp. longum JCM 1217
**VII**	B. bifidum BGN4<br>B. bifidum JCM 1255<br>B. bifidum ATCC 29521<br>B. bifidum PRL2010
**VIII**	B. adolescentis ATCC 15703
**IX**	B. angulatum JCM 7096
**X**	B. breve JCM 7017<br>B. breve JCM 1192
**XI**	B. breve ACS-071-V-Sch8b
**XII**	B. adolescentis BBMN23

**XIII**	B. breve JCM 7019
**XIV**	B. longum subsp. infantis 157F<br>B. longum DJO10A<br>B. longum subsp. longum GT15<br>B. longum subsp. longum KACC 91563
**XV**	B. kashiwanohense JCM 15439
**XVI**	B. longum subsp. infantis ATCC 15697
**XVII**	B. longum subsp. longum JDM301<br>B. longum BXY01
**XVIII**	B. longum subsp. infantis ATCC 15697<br>B. longum subsp. infantis JCM 1222
**XIX**	B. longum subsp. longum KACC 91563
**XX**	B. bifidum PRL2010
**XXI**	B. kashiwanohense PV20-2
**XXII**	B. breve JCM 7019<br>B. breve ACS-071-V-Sch8b<br>B. breve UCC2003<br>B. longum subsp. longum JDM301<br>B. breve 689b<br>B. longum BXY01
**XXIII**	B. breve NCFB 2258<br>B. breve JCM 7017<br>B. breve ACS-071-V-Sch8b<br>B. breve JCM 1192<br>B. breve S27<br>B. breve 12L
**XXIV**	B. breve NCFB 2258<br>B. breve JCM 7017<br>B. breve ACS-071-V-Sch8b<br>B. breve JCM 1192
**XXV**	B. breve NCFB 2258
**XXVI**	B. bifidum JCM 1255<br>B. bifidum ATCC 29521
**XXVII**	B. longum subsp. longum BBMN68<br>B. longum subsp. longum GT15<br>B. longum subsp. longum KACC 91563
**XXVIII**	B. thermophilum RBL67
**XXIX**	B. longum 105-A<br>B. longum subsp. longum JCM 1217
**XXX**	B. breve ACS-071-V-Sch8b<br>B. breve 12L
**RM3**	
**I**	B. longum subsp. infantis 157F<br>B. longum DJO10A<br>B. longum NCC2705<br>B. longum 105-A<br>B. longum subsp. longum JCM 1217
**II**	B. longum subsp. infantis 157F
**III**	B. longum subsp. infantis ATCC 15697<br>B. longum subsp. infantis JCM 1222
**IV**	B. breve JCM 7019<br>B. longum subsp. infantis ATCC 15697

	B. longum subsp. infantis JCM 1222
**V**	B. longum subsp. infantis 157F<br>B. longum DJO10A<br>B. longum NCC2705<br>B. longum subsp. longum GT15<br>B. longum subsp. longum KACC 91563
**VI**	B. longum subsp. infantis 157F<br>B. longum DJO10A
**VII**	B. longum subsp. longum BBMN68<br>B. longum 105-A<br>B. longum subsp. longum KACC 91563<br>B. longum subsp. longum JCM 1217
**VIII**	B. longum subsp. longum JDM301
**IX**	B. longum subsp. longum JDM301<br>B. longum strain BXY01
**X**	B. longum subsp. infantis ATCC 15697
**XI**	B. breve NCFB 2258<br>B. breve JCM 7017<br>B. breve ACS-071-V-Sch8b<br>B. breve JCM 1192<br>B. longum subsp. infantis 157F<br>B. breve S27<br>B. longum subsp. longum KACC 91563<br>B. breve 12L
**XII**	B. adolescentis BBMN23
**XIII**	B. kashiwanohense JCM 15439
**XIV**	B. longum subsp. longum BBMN68<br>B. longum subsp. longum KACC 91563
**XV**	B. longum subsp. longum KACC 91563
**XVI**	B. longum BXY01<br>B. longum subsp. longum JDM301
**XVII**	B. longum subsp. infantis 157F<br>B. longum DJO10A<br>B. longum subsp. longum GT15<br>B. longum subsp. longum KACC 91563
**XVIII**	B. pseudocatenulatum JCM 1200
**XIX**	B. kashiwanohense PV20-2
**XX**	B. breve NCFB 2258<br>B. breve JCM 7017<br>B. breve JCM 1192<br>B. breve S27
**RM4**	
**I**	B. longum subsp. infantis ATCC 15697
**II**	B. adolescentis BBMN23
**III**	B. longum subsp. longum JDM301<br>B. longum BXY01
**IV**	B. adolescentis ATCC 15703
**V**	B. longum subsp. longum KACC 91563
**VI**	B. thermophilum RBL67
**VII**	B. bifidum S17<br>B. bifidum BGN4

	B. bifidum JCM 1255<br>B. bifidum ATCC 29521
**VIII**	B. longum subsp. longum JDM301
**IX**	B. longum strain BXY01<br>B. longum subsp. longum JDM301
**X**	B. kashiwanohense JCM 15439
**RM5**	
**I**	B. adolescentis BBMN23
**II**	B. adolescentis ATCC 15703
