# Supplementary material for: Employing toxin-antitoxin genome markers for identification of Bifidobacterium and Lactobacillus strains in human metagenomes
Source: PeerJ. 2019 Mar 4;7:e6554. doi: 10.7717/peerj.6554 (PMC6404652; doi:10.7717/peerj.6554)
Supplement: Supplemental Information 2 — Strain diversity of Lactobacillus in metagenomes. [file peerj-07-6554-s002.pdf]

Table S2 Strain diversity of *Lactobacillus* in metagenomes

Groups	Representatives
**RM1**	
**I**	L. johnsonii N6.2
**II**	L. rhamnosus Lc 705<br>L. rhamnosus LOCK908<br>L. rhamnosus ATCC 8530
**RM2**	
**I**	L. rhamnosus ATCC 8530<br>L. rhamnosus LOCK908<br>L. rhamnosus Lc 705
**II**	L. paracasei N1115<br>L. casei BL23<br>L. casei LOCK919<br>L. casei LC2W<br>L. casei W56<br>L. casei BD-II<br>L. casei str. Zhang<br>L. casei 12A<br>L. paracasei subsp. paracasei JCM 8130<br>L. paracasei ATCC 334<br>L. paracasei subsp. paracasei 8700:2
**III**	L. buchneri NRRL B-30929<br>L. buchneri CD034
**RM4**	
**I**	L. rhamnosus ATCC 8530<br>L. rhamnosus LOCK908<br>L. rhamnosus Lc 705
**II**	L. sanfranciscensis TMW 1.1304
**III**	L. paracasei N1115<br>L. casei BL23<br>L. casei LOCK919<br>L. casei LC2W<br>L. casei W56<br>L. casei str. Zhang<br>L. casei BD-II<br>L. casei 12A<br>L. paracasei subsp. paracasei JCM 8130<br>L. paracasei ATCC 334<br>L. paracasei subsp. paracasei 8700:2
**RM5**	

**I**	L. paracasei N1115<br>L. casei BL23<br>L. casei LOCK919<br>L. casei LC2W<br>L. casei W56<br>L. casei BD-II<br>L. casei str. Zhang<br>L. casei 12A<br>L. paracasei subsp. paracasei JCM 8130<br>L. paracasei ATCC 334<br>L. paracasei subsp. paracasei 8700:2
**II**	L. buchneri NRRL B-30929<br>L. buchneri CD034
**III**	L. ruminis ATCC 27782
