# Supplementary material for: Employing toxin-antitoxin genome markers for identification of Bifidobacterium and Lactobacillus strains in human metagenomes
Source: PeerJ. 2019 Mar 4;7:e6554. doi: 10.7717/peerj.6554 (PMC6404652; doi:10.7717/peerj.6554)
Supplement: Supplemental Information 3 — Strain diversity of Lactobacillus and Bifidobacterium in the samples of the intestinal metagenomes (from the Human Microbiome Project database). [file peerj-07-6554-s003.pdf]

Table S3 Strain diversity of *Lactobacillus* and *Bifidobacterium* in the samples of the intestinal metagenomes (from the Human Microbiome Project database)

Project	*Lactobacillus sp.*	*Bifidobacterium sp.*
SRS011239	-	B. longum DJO10A 1/1[1] [278/363][2]<br>B. longum subsp. infantis 157F 2/5 [278/363, 210/219]<br>B. longum subsp. longum KACC 91563 2/4 [116/393, 219/219]<br>B. adolescentis BBMN23 3/4 [309/309, 182/291, 300/300]<br>B. adolescentis ATCC 15703 1/1 [505/519]
SRS011271	-	B. adolescentis BBMN23 3/4 [309/309, 136/291, 267/300]
SRS011405	-	B. adolescentis BBMN23 2/4 [106/309, 93/300]
SRS011452		B. longum subsp. infantis 157F 3/5 [255/285, 28/363, 36/336] 0<br>B. breve JCM 7019 2/7 [313/369, 115/285]
SRS011529	L. ruminis ATCC 27782 3/4 [236/273, 285/384, 119/288]	-
SRS012273	L. paracasei ATCC 334 2/3 [180/384, 21/270]	B. longum subsp. infantis 157F 2/5 [24/270, 246/363]<br>B. longum DJO10A 1/1 [246/363]<br>B. kashiwanohense JCM 15439 5/11 [28/324, 158/291, 232/309, 21/270, 24/294]<br>B. longum subsp. longum GT15 1/1 [301/363]<br>B. longum subsp. longum BBMN68 1/1 [281/393]<br>B. longum 105-A 1/1 [297/363]<br>B. adolescentis BBMN23 4/4 [65/309, 232/246, 269/291, 246/300]
SRS012902		B. pseudocatenulatum JCM 1200 2/2 [318/665, 285/330]<br>B. kashiwanohense JCM 15439 3/11 [192/297, 98/270, 110/330]<br>B. adolescentis BBMN23 1/4 [78/291]
SRS013158		B. breve JCM 1192 3/7 [124/264, 287/324, 83/294]<br>B. breve S27 2/7 [309/324, 83/294]<br>B. breve JCM 7017 3/9 [130/270, 83/294, 322/324]<br>B. breve NCFB 2258 4/10 [83/294, 36/294, 227/264, 304/324]<br>B. breve JCM 7019 5/7 [131/294, 124/264, 184/294, 252/324, 126/357]<br>B. breve 689b 5/5 [83/294, 169/324, 124/264, 126/357, 131/294]<br>B. longum subsp. infantis JCM 1222 6/14 [101/294, 221/324, 83/294, 52/357, 197/363, 62/270]

		B. longum subsp. longum BBMN68 1/1 [393/393]<br>B. longum subsp. infantis 157F 4/5 [270/270, 184/363, 228/336, 90/219]<br>B. longum subsp. longum JCM 1217 1/1 [378/393]<br>B. longum subsp. longum KACC 91563 2/4 [311/393, 165/219]<br>B. longum subsp. infantis ATCC 15697 6/13 [83/294, 101/294, 56/264, 52/354, 197/363, 221/324]<br>B. longum 105-A 1/1 [293/363]<br>B. kashiwanohense JCM 15439 3/11 [305/324, 24/294, 83/330]<br>B. breve ACS-071-V-Sch8b 4/9 [101/294, 83/294, 287/324, 179/270]<br>B. bifidum BGN4 3/4 [38/180, 66/180, 228/324]<br>B. bifidum PRL2010 3/4 [228/324, 23/375, 38/180]<br>B. bifidum JCM 1255 3/4 [23/360, 228/324, 38/180]<br>B. bifidum ATCC 29521 3/4 [207/303, 23/360, 38/180]<br>B. longum BXY01 4/8 [131/294, 237/363, 322/324, 179/270]<br>B. longum subsp. longum JDM301 6/11 [131/294, 74/357, 21/285, 322/324, 209/300, 237/363]<br>B. breve 12L 3/6 [83/294, 74/357, 322/324]<br>B. pseudocatenulatum JCM 1200 2/2 [112/665, 292/330]<br>B. breve UCC2003 4/7 [130/270, 131/294, 318/324, 83/294]
SRS013215		B. longum subsp. longum KACC 91563 2/4 [72/393, 219/219]<br>B. breve NCFB 2258 2/10 [132/264, 37/276]<br>B. longum subsp. infantis 157F 3/5 [206/270, 186/363, 193/219]<br>B. pseudocatenulatum JCM 1200 1/2 [277/330]
SRS013476		B. adolescentis BBMN23 4/4 [309/309, 235/246, 262/291, 300/300]<br>B. kashiwanohense JCM 15439 3/11 [187/291, 73/309, 24/294]
SRS013951		B. breve UCC2003 2/7 [160/270, 148/294]<br>B. breve JCM 7019 2/7 [199/294, 154/264]<br>B. longum subsp. longum JDM301 3/11 [199/294, 190/300, 329/363]<br>B. longum subsp. infantis 157F 3/5 [160/270, 363/363, 336/336]<br>B. longum DJO10A 1/1 [363/363]<br>B. longum subsp. longum KACC 91563 2/4 [307/393, 38/219]

		B. longum BXY01 3/8 [199/294, 329/363, 160/270]<br>B. longum subsp. infantis JCM 1222 3/14 [146/294, 241/363, 53/270]<br>B. longum subsp. infantis ATCC 15697 3/13 [146/294, 47/264, 241/363]<br>B. bifidum BGN4 1/4 [189/297]<br>B. adolescentis ATCC 15703 1/1 [263/519]<br>B. adolescentis BBMN23 2/4 [178/246, 68/291]
SRS014313		B. longum subsp. longum JDM301 4/11 [107/246, 297/321, 339/363, 61/324]<br>B. longum subsp. longum BBMN68 1/1 [224/393]<br>B. longum BXY01 3/8 [339/363, 297/321, 107/246]
SRS014683		B. bifidum BGN4 4/4 [112/180, 31/180, 297/297, 143/324]<br>B. bifidum ATCC 29521 4/4 [227/297, 119/303, 360/360, 112/180]<br>B. bifidum S17 4/4 [112/180, 271/324, 294/375, 227/297]<br>B. bifidum PRL2010 4/4 [224/297, 143/324, 344/375, 180/180]<br>B. bifidum JCM 1255 4/4 [360/360, 227/297, 143/324, 112/180]<br>B. longum subsp. longum JDM301 4/11 [101/186, 285/285, 159/324, 263/363]<br>B. longum BXY01 3/8 [263/363, 101/186, 159/324]<br>B. longum subsp. infantis ATCC 15697 3/13 [38/348, 285/363, 115/324]<br>B. longum subsp. longum KACC 91563 2/4 [302/393, 219/219]<br>B. longum subsp. longum JCM 1217 1/1 [329/393]<br>B. longum DJO10A 1/1 [341/363]<br>B. longum subsp. infantis 157F 2/5 [341/363, 215/219]<br>B. longum subsp. longum BBMN68 1/1 [322/393]<br>B. longum 105-A 1/1 [308/363]<br>B. angulatum JCM 7096 2/2 [294/294, 301/318]<br>B. adolescentis ATCC 15703 1/1 [518/519]<br>B. pseudocatenulatum JCM 1200 2/2 [126/665, 215/330]<br>B. adolescentis BBMN23 3/4 [143/309, 165/291, 300/300]
SRS014923		B. breve 12L 3/6 [24/276, 22/357, 196/324]<br>B. breve UCC2003 4/7 [106/270, 22/357, 192/294, 196/324]<br>B. longum strain BXY01 2/3 [285/285, 22/357]<br>B. longum subsp. longum JDM301 7/11

		[211/294, 168/186, 22/357, 285/285, 196/324, 106/300, 129/363]<br>B. longum subsp. longum BBMN68 1/1 [214/393]<br>B. longum subsp. infantis JCM 1222 6/14 [167/294, 356/369, 113/285, 26/324, 22/357, 51/363]<br>B. breve 689b 4/5 [135/324, 106/264, 22/357, 192/294]<br>B. longum subsp. infantis 157F 4/5 [155/270, 261/285, 51/363, 275/336]<br>B. breve JCM 7019 6/7 [369/369, 211/294, 106/264, 129/285, 135/324, 22/357]<br>B. longum BXY01 5/8 [211/294, 129/363, 168/186, 196/324, 106/270]<br>B. longum subsp. infantis ATCC 15697 6/13 [167/294, 22/354, 51/363, 356/369, 26/324, 113/285]<br>B. pseudocatenulatum JCM 1200 2/2 [228/665, 167/330]<br>B. bifidum BGN4 3/4 [135/180, 24/180, 314/324]<br>B. bifidum PRL2010 2/4 [314/324, 135/180]<br>B. bifidum JCM 1255 2/4 [314/324, 135/180]<br>B. bifidum ATCC 29521 2/4 [294/303, 135/180]<br>B. adolescentis BBMN23 3/4 [182/246, 291/291, 22/300]
SRS015133		B. longum NCC2705 1/1 [237/363]<br>B. adolescentis BBMN23 1/4 [182/291]
SRS015190		B. longum subsp. longum KACC 91563 2/4 [130/393, 150/219]<br>B. longum subsp. infantis 157F 3/5 [265/363, 21/336, 219/219]<br>B. longum DJO10A 1/1 [265/363]<br>B. longum subsp. longum JCM 1217 1/1 [210/393]
SRS015264		B. longum subsp. infantis 157F 2/5 [100/270, 252/363]<br>B. longum DJO10A 1/1 [252/363]<br>B. longum NCC2705 1/1 [200/363]
SRS015578		B. longum strain BXY01 1/3 [215/285]<br>B. longum subsp. longum KACC 91563 2/4 [242/393, 213/219]<br>B. longum subsp. longum JDM301 3/11 [21/294, 215/285, 57/363]<br>B. longum subsp. infantis 157F 3/5 [157/363, 21/336, 190/219]<br>B. adolescentis 22L 1/1 [130/180]<br>B. adolescentis ATCC 15703 1/1 [519/519]<br>B. adolescentis BBMN23 1/4 [182/291]
SRS015663		B. adolescentis ATCC 15703 1/1 [323/519]<br>B. pseudocatenulatum JCM 1200 1/2

		[271/330]<br>B. adolescentis BBMN23 1/4 [153/291]
SRS015854		B. adolescentis BBMN23 4/4 [131/309, 91/246, 116/291, 300/300]
SRS016267		B. longum subsp. longum KACC 91563 2/4 [128/393, 170/219]
SRS016495		B. breve ACS-071-V-Sch8b 4/9 [283/294, 160/324, 37/276, 180/270]<br>B. longum subsp. longum JDM301 4/11 [294/294, 226/324, 207/300, 354/363]<br>B. breve UCC2003 3/7 [199/270, 294/294, 160/324]<br>B. breve JCM 1192 4/7 [37/276, 193/264, 118/324, 214/294]<br>B. breve NCFB 2258 5/10 [144/171, 163/294, 250/264, 75/324, 37/276]<br>B. longum subsp. infantis 157F 5/5 [270/270, 285/285, 363/363, 336/336, 218/219]<br>B. longum DJO10A 1/1 [363/363]<br>B. breve 689b 3/5 [117/324, 193/264, 294/294]<br>B. breve JCM 7017 4/9 [199/270, 214/294, 184/324, 37/276]<br>B. breve JCM 7019 5/7 [369/369, 294/294, 193/264, 116/285, 160/324]<br>B. longum BXY01 4/8 [294/294, 354/363, 226/324, 177/270]<br>B. longum subsp. infantis JCM 1222 7/14 [286/294, 368/369, 41/141, 107/285, 115/324, 346/363, 239/270]<br>B. longum subsp. infantis ATCC 15697 6/13 [286/294, 233/264, 346/363, 368/369, 115/324, 107/285]<br>B. kashiwanohense JCM 15439 7/11 [223/291, 115/309, 160/324, 63/270, 24/294, 60/330, 24/318]<br>B. bifidum BGN4 3/4 [97/180, 47/180, 313/324]<br>B. bifidum JCM 1255 2/4 [313/324, 97/180]<br>B. bifidum ATCC 29521 2/4 [292/303, 97/180]<br>B. longum NCC2705 1/1 [362/363]<br>B. longum subsp. longum BBMN68 1/1 [393/393]<br>B. pseudocatenulatum JCM 1200 2/2 [142/665, 323/330]<br>B. adolescentis BBMN23 4/4 [25/309, 231/246, 147/291, 136/300]<br>B. longum subsp. longum JCM 1217 1/1 [393/393]<br>B. longum 105-A 1/1 [355/363]<br>B. longum subsp. longum KACC 91563 3/4 [393/393, 21/360, 219/219]<br>B. breve 12L 2/6 [37/276, 226/324]
SRS016517		B. longum subsp. longum JDM301 5/11 [294/294, 25/294, 60/324, 209/300,

		361/363]<br>B. breve UCC2003 5/7 [199/270, 294/294, 23/246, 60/324, 25/294]<br>B. breve JCM 1192 5/7 [37/276, 193/264, 60/324, 203/294, 25/294]<br>B. breve NCFB 2258 7/10 [25/294, 144/171, 23/246, 166/294, 253/264, 36/324, 37/276]<br>B. breve ACS-071-V-Sch8b 6/9 [291/294, 25/294, 60/324, 37/276, 180/270, 23/246]<br>B. longum subsp. infantis 157F 5/5 [270/270, 284/285, 363/363, 336/336, 219/219]<br>B. longum subsp. infantis JCM 1222 8/14 [286/294, 360/369, 112/285, 24/324, 25/294, 359/363, 191/270, 23/246]<br>B. longum DJO10A 1/1 [363/363]<br>B. breve 689b 4/5 [25/294, 60/324, 193/264, 294/294]<br>B. breve JCM 7017 6/9 [199/270, 203/294, 23/246, 25/294, 60/324, 37/276]<br>B. breve JCM 7019 6/7 [369/369, 294/294, 193/264, 25/294, 130/285, 60/324]<br>B. longum BXY01 5/8 [25/294, 294/294, 361/363, 60/324, 179/270]<br>B. longum subsp. infantis ATCC 15697 8/13 [25/294, 286/294, 185/264, 359/363, 360/369, 23/252, 24/324, 112/285]<br>B. bifidum PRL2010 4/4 [40/297, 114/324, 234/375, 135/180]<br>B. bifidum S17 4/4 [168/180, 36/324, 235/375, 254/297]<br>B. bifidum BGN4 4/4 [168/180, 126/180, 137/297, 114/324]<br>B. bifidum JCM 1255 4/4 [220/360, 254/297, 114/324, 168/180]<br>B. bifidum ATCC 29521 4/4 [254/297, 78/303, 220/360, 168/180]<br>B. kashiwanohense JCM 15439 7/11 [48/246, 158/291, 60/309, 60/324, 174/270, 29/294, 53/330]<br>B. longum NCC2705 1/1 [362/363]<br>B. longum subsp. longum GT15 1/1 [362/363]<br>B. longum subsp. longum KACC 91563 2/4 [393/393, 219/219]<br>B. longum subsp. longum BBMN68 1/1 [390/393]<br>B. adolescentis BBMN23 4/4 [23/309, 206/246, 218/291, 91/300]<br>B. longum subsp. longum JCM 1217 1/1 [390/393]<br>B. pseudocatenulatum JCM 1200 2/2

		[314/665, 330/330]
SRS016753		B. bifidum S17 1/4 [244/324]<br>B. breve JCM 7019 2/7 [101/294, 244/324]<br>B. longum subsp. longum JDM301 5/11 [101/294, 126/186, 261/285, 143/324, 118/363]<br>B. longum strain BXY01 1/3 [261/285]<br>B. longum BXY01 4/8 [101/294, 118/363, 126/186, 143/324]
SRS017103	L. acidophilus La-14 4/4 [170/309, 279/279, 348/348, 335/342] 2<br>L. acidophilus FSI4 4/4 [348/348, 335/342, 279/279, 170/309] 2<br>L. acidophilus NCFM 4/4 [170/309, 279/279, 335/342, 348/348] 2	B. longum subsp. longum JDM301 3/11 [21/294, 21/285, 242/363]<br>B. longum subsp. longum GT15 1/1 [288/363]<br>B. breve NCFB 2258 2/10 [171/171, 21/294]<br>B. longum subsp. infantis 157F 3/5 [253/363, 21/336, 184/219]<br>B. longum subsp. infantis JCM 1222 1/14 [221/363]<br>B. longum DJO10A 1/1 [253/363]<br>B. longum NCC2705 1/1 [253/363]<br>B. longum subsp. longum BBMN68 1/1 [375/393]<br>B. longum subsp. longum KACC 91563 2/4 [393/393, 219/219]<br>B. adolescentis BBMN23 3/4 [246/246, 291/291, 22/300]<br>B. adolescentis ATCC 15703 1/1 [519/519]
SRS017247		B. kashiwanohense JCM 15439 4/11 [65/291, 166/309, 181/270, 91/330]<br>B. pseudocatenulatum JCM 1200 2/2 [145/665, 177/330]
SRS017521		B. longum subsp. longum JDM301 3/11 [186/186, 285/285, 208/363]<br>B. longum subsp. infantis 157F 2/5 [307/363, 153/219]<br>B. longum subsp. longum JCM 1217 1/1 [308/393]<br>B. longum subsp. longum GT15 1/1 [262/363]<br>B. longum BXY01 2/8 [208/363, 186/186]<br>B. longum subsp. longum BBMN68 1/1 [284/393]<br>B. longum 105-A 1/1 [363/363]<br>B. adolescentis BBMN23 3/4 [160/309, 182/291, 300/300]<br>B. longum subsp. longum KACC 91563 2/4 [187/393, 219/219]
SRS017701		B. pseudocatenulatum JCM 1200 2/2 [318/665, 330/330]
SRS018133		B. longum subsp. longum JDM301 3/11 [145/294, 69/300, 284/363]<br>B. longum subsp. infantis 157F 4/5 [130/270, 308/363, 216/336, 191/219]<br>B. longum DJO10A 1/1 [308/363]

		B. longum subsp. longum JCM 1217 1/1 [384/393]<br>B. breve JCM 7019 3/7 [369/369, 145/294, 33/264]<br>B. longum NCC2705 1/1 [308/363]<br>B. longum BXY01 3/8 [145/294, 284/363, 39/270]<br>B. longum subsp. infantis JCM 1222 4/14 [105/294, 353/369, 234/363, 39/270]<br>B. longum subsp. infantis ATCC 15697 5/13 [105/294, 33/264, 38/348, 234/363, 353/369]<br>B. longum subsp. longum BBMN68 1/1 [384/393]<br>B. longum subsp. longum KACC 91563 2/4 [352/393, 219/219]
SRS018313		B. longum subsp. longum JCM 1217 1/1 [223/393]<br>B. longum subsp. longum KACC 91563 1/4 [318/393]<br>B. longum subsp. longum BBMN68 1/1 [315/393]
SRS018351		B. longum BXY01 3/8 [121/294, 224/363, 36/270]<br>B. longum subsp. longum JDM301 3/11 [121/294, 36/300, 224/363]<br>B. longum subsp. infantis 157F 4/5 [72/270, 286/363, 289/336, 159/219]<br>B. longum DJO10A 1/1 [286/363]<br>B. longum subsp. longum JCM 1217 1/1 [247/393]<br>B. longum NCC2705 1/1 [286/363]<br>B. longum subsp. longum KACC 91563 2/4 [268/393, 219/219]<br>B. longum subsp. longum BBMN68 1/1 [247/393]<br>B. adolescentis BBMN23 3/4 [285/309, 154/291, 300/300]<br>B. adolescentis ATCC 15703 1/1 [519/519]
SRS018427		B. longum subsp. longum KACC 91563 1/4 [247/393]<br>B. longum subsp. longum JDM301 3/11 [212/294, 153/300, 282/363]<br>B. breve UCC2003 2/7 [123/270, 163/294]<br>B. longum subsp. longum GT15 1/1 [304/363]<br>B. breve JCM 1192 2/7 [117/264, 21/294]<br>B. breve NCFB 2258 2/10 [105/294, 180/264]<br>B. longum subsp. infantis 157F 3/5 [249/270, 363/363, 310/336]<br>B. longum DJO10A 1/1 [363/363]<br>B. breve 689b 2/5 [117/264, 163/294]<br>B. longum NCC2705 1/1 [319/363]

		B. longum subsp. longum JCM 1217 1/1 [288/393]<br>B. breve JCM 7019 3/7 [82/369, 212/294, 117/264]<br>B. longum BXY01 3/8 [212/294, 282/363, 123/270]<br>B. longum subsp. longum BBMN68 1/1 [221/393]<br>B. longum 105-A 1/1 [323/363] 3<br>B. longum subsp. infantis JCM 1222 4/14 [149/294, 82/369, 182/363, 54/270]<br>B. longum subsp. infantis ATCC 15697 4/13 [149/294, 48/264, 182/363, 82/369]
SRS018656		B. breve ACS-071-V-Sch8b 2/9 [157/294, 151/270]<br>B. longum subsp. longum JDM301 3/11 [215/294, 181/300, 94/363]<br>B. longum subsp. infantis ATCC 15697 4/13 [159/294, 41/264, 38/348, 131/363]<br>B. longum subsp. infantis 157F 3/5 [270/270, 193/363, 336/336]<br>B. longum subsp. infantis JCM 1222 3/14 [159/294, 131/363, 47/270]<br>B. breve 689b 2/5 [145/264, 213/294]<br>B. longum subsp. longum JCM 1217 1/1 [235/393]<br>B. breve UCC2003 2/7 [151/270, 213/294]<br>B. breve JCM 7019 2/7 [215/294, 145/264]<br>B. longum BXY01 3/8 [215/294, 94/363, 151/270]<br>B. longum subsp. longum BBMN68 1/1 [235/393]<br>B. pseudocatenulatum JCM 1200 2/2 [298/665, 300/330]
SRS019030		B. longum subsp. longum JDM301 5/11 [21/294, 129/186, 285/285, 156/324, 246/363]<br>B. longum BXY01 4/8 [21/294, 246/363, 129/186, 156/324]<br>B. adolescentis ATCC 15703 1/1 [518/519]<br>B. longum subsp. infantis 157F 3/5 [255/363, 21/336, 100/219]<br>B. longum DJO10A 1/1 [255/363]<br>B. longum NCC2705 1/1 [255/363]<br>B. longum subsp. longum JCM 1217 1/1 [290/393]<br>B. longum subsp. longum GT15 1/1 [255/363]<br>B. longum subsp. longum BBMN68 1/1 [290/393]<br>B. adolescentis BBMN23 3/4 [205/309, 160/291, 300/300]

		B. longum subsp. longum KACC 91563 2/4 [339/393, 100/219]
SRS019161		B. longum subsp. longum JDM301 3/11 [99/294, 82/300, 272/363]<br>B. longum subsp. longum BBMN68 1/1 [275/393]<br>B. longum subsp. infantis 157F 3/5 [122/270, 363/363, 184/336]<br>B. longum DJO10A 1/1 [363/363]<br>B. longum NCC2705 1/1 [296/363]<br>B. longum subsp. longum JCM 1217 1/1 [266/393]<br>B. longum 105-A 1/1 [321/363]<br>B. longum BXY01 3/8 [99/294, 272/363, 52/270]<br>B. longum subsp. longum GT15 1/1 [221/363]<br>B. adolescentis BBMN23 2/4 [236/246, 109/291] 0<br>B. longum subsp. longum KACC 91563 2/4 [35/297, 247/393]
SRS019601		B. breve ACS-071-V-Sch8b 7/9 [244/255, 160/294, 289/294, 183/276, 41/357, 154/270, 71/246]<br>B. breve UCC2003 5/7 [154/270, 41/357, 106/294, 71/246, 289/294]<br>B. breve JCM 1192 6/7 [41/354, 183/276, 148/264, 244/255, 160/294, 289/294]<br>B. breve NCFB 2258 7/10 [244/255, 289/294, 71/246, 250/294, 148/264, 41/357, 183/276]<br>B. breve S27 5/7 [41/357, 244/255, 183/276, 71/246, 246/294]<br>B. longum subsp. infantis JCM 1222 6/14 [107/294, 249/294, 187/357, 53/270, 21/333, 71/246]<br>B. breve 689b 4/5 [289/294, 148/264, 187/357, 106/294]<br>B. breve 12L 5/6 [193/294, 183/276, 71/246, 146/357, 244/255]<br>B. breve JCM 7017 7/9 [244/255, 154/270, 41/357, 160/294, 71/246, 289/294, 183/276]<br>B. breve JCM 7019 4/7 [107/294, 148/264, 249/294, 187/357]<br>B. longum subsp. infantis ATCC 15697 6/13 [249/294, 107/294, 47/264, 21/348, 184/354, 71/252]
SRS019910		B. pseudocatenulatum JCM 1200 2/2 [297/665, 312/330]
SRS019968		B. adolescentis BBMN23 4/4 [103/309, 199/246, 131/291, 264/300]<br>B. adolescentis ATCC 15703 1/1 [433/519]
SRS020233		B. adolescentis BBMN23 4/4 [309/309, 246/246, 274/291, 300/300]
SRS020328		B. bifidum PRL2010 4/4 [92/297,

		324/324, 260/375, 150/180]<br>B. longum BXY01 4/8 [115/294, 301/363, 224/324, 112/270]<br>B. longum subsp. longum JDM301 5/11 [115/294, 21/285, 224/324, 142/300, 301/363]<br>B. bifidum S17 4/4 [54/180, 136/324, 307/375, 181/297]<br>B. breve 689b 3/5 [163/324, 106/264, 115/294]<br>B. breve NCFB 2258 2/10 [213/264, 111/324]<br>B. breve UCC2003 3/7 [112/270, 115/294, 223/324]<br>B. bifidum BGN4 4/4 [54/180, 173/180, 213/297, 324/324]<br>B. bifidum JCM 1255 4/4 [321/360, 181/297, 324/324, 54/180]<br>B. breve 12L 1/6 [224/324]<br>B. breve JCM 7017 2/9 [112/270, 199/324]<br>B. bifidum ATCC 29521 4/4 [181/297, 303/303, 321/360, 54/180]<br>B. longum subsp. infantis 157F 4/5 [270/270, 361/363, 261/336, 179/219]<br>B. longum DJO10A 1/1 [361/363]<br>B. longum subsp. longum JCM 1217 1/1 [254/393]<br>B. longum 105-A 1/1 [258/363]<br>B. adolescentis BBMN23 4/4 [196/309, 246/246, 284/291, 300/300]<br>B. longum subsp. longum KACC 91563 2/4 [162/393, 219/219]<br>B. adolescentis ATCC 15703 1/1 [518/519]
SRS020869	L. ruminis ATCC 27782 3/4 [268/273, 384/384, 288/288] 0	B. breve NCFB 2258 3/10 [28/324, 105/294, 231/264]<br>B. longum subsp. infantis ATCC 15697 4/13 [207/294, 54/264, 220/363, 21/324]<br>B. longum subsp. infantis 157F 3/5 [270/270, 323/363, 336/336]<br>B. longum DJO10A 1/1 [323/363]<br>B. breve UCC2003 3/7 [168/270, 187/294, 28/324]<br>B. longum subsp. longum BBMN68 1/1 [308/393]<br>B. longum subsp. longum JDM301 5/11 [210/294, 21/285, 198/300, 288/363, 28/324]<br>B. longum subsp. infantis JCM 1222 4/14 [207/294, 21/324, 220/363, 60/270]<br>B. longum 105-A 1/1 [356/363]<br>B. longum BXY01 3/8 [210/294, 288/363, 168/270]<br>B. longum subsp. longum JCM 1217 1/1 [301/393]

		B. breve 689b 2/5 [162/264, 187/294]<br>B. animalis subsp. lactis B420 5/5 [54/270, 237/327, 172/231, 97/255, 165/165]<br>B. animalis subsp. lactis DSM 10140 5/5 [54/270, 283/384, 172/231, 103/261, 236/306]<br>B. animalis subsp. lactis BLC1 5/5 [237/327, 172/231, 103/261, 283/384, 54/270]<br>B. animalis subsp. lactis Bi-07 5/5 [54/270, 97/255, 165/165, 172/231, 236/306]<br>B. animalis subsp. lactis Bl12 5/5 [172/231, 283/384, 237/327, 54/270, 103/261]<br>B. animalis subsp. lactis KLDS2.0603 4/4 [236/306, 172/231, 103/261, 283/384]<br>B. animalis subsp. lactis AD011 4/4 [191/261, 54/270, 103/261, 172/195]<br>B. animalis subsp. lactis BB-12 5/5 [172/231, 356/615, 93/309, 82/204, 237/327]<br>B. animalis RH 6/6 [172/231, 54/270, 103/261, 283/384, 54/270, 236/306]<br>B. animalis subsp. lactis CNCM I-2494 4/4 [356/615, 93/309, 172/231, 237/327]<br>B. animalis A6 5/5 [103/261, 54/270, 165/165, 172/231, 236/306]<br>B. animalis subsp. lactis V9 5/5 [236/306, 172/231, 283/384, 103/261, 54/270]<br>B. animalis subsp. lactis Bl-04 5/5 [236/306, 103/261, 172/231, 283/384, 54/270]<br>B. adolescentis BBMN23 1/4 [182/291]<br>B. animalis subsp. animalis ATCC 25527 4/7 [34/270, 151/261, 36/135, 29/282]
SRS021948		B. longum subsp. longum BBMN68 1/1 [246/393]<br>B. longum subsp. longum JCM 1217 1/1 [246/393]
SRS022071		B. longum subsp. infantis 157F 2/5 [262/363, 21/336]<br>B. longum subsp. infantis JCM 1222 1/14 [237/363]<br>B. longum DJO10A 1/1 [262/363]<br>B. longum subsp. longum JCM 1217 1/1 [290/393]<br>B. longum 105-A 1/1 [331/363]<br>B. longum subsp. longum BBMN68 1/1 [308/393]<br>B. longum subsp. longum GT15 1/1 [304/363]<br>B. kashiwanohense JCM 15439 3/11

		[143/291, 240/309, 24/294]<br>B. adolescentis BBMN23 4/4 [156/309, 246/246, 291/291, 274/300]
SRS022137		B. longum strain BXY01 1/3 [285/285]<br>B. longum subsp. longum JDM301 2/11 [285/285, 28/363]<br>B. adolescentis ATCC 15703 1/1 [245/519]
SRS022713		B. breve UCC2003 3/7 [95/246, 200/324, 84/324]<br>B. kashiwanohense JCM 15439 3/11 [23/318, 200/324, 84/324]
SRS023526		B. adolescentis BBMN23 3/4 [309/309, 182/291, 300/300]
SRS023583		B. longum subsp. infantis JCM 1222 2/14 [23/369, 202/363]<br>B. longum subsp. infantis ATCC 15697 2/13 [202/363, 23/369]<br>B. longum subsp. longum KACC 91563 1/4 [233/393]<br>B. longum subsp. longum JDM301 2/11 [122/285, 214/363]<br>B. longum subsp. longum JCM 1217 1/1 [302/393]<br>B. longum DJO10A 1/1 [273/363]<br>B. longum NCC2705 1/1 [234/363]<br>B. longum subsp. infantis 157F 1/5 [277/363]<br>B. longum BXY01 1/8 [214/363]<br>B. longum subsp. longum BBMN68 1/1 [298/393]<br>B. longum 105-A 1/1 [238/363]
SRS023829		B. pseudocatenulatum JCM 1200 2/2 [301/665, 223/330]
SRS023971		B. bifidum BGN4 3/4 [44/180, 86/180, 238/324]<br>B. bifidum PRL2010 2/4 [238/324, 91/180]<br>B. bifidum JCM 1255 2/4 [238/324, 44/180]<br>B. bifidum ATCC 29521 2/4 [217/303, 44/180]<br>B. longum subsp. longum KACC 91563 2/4 [290/393, 212/219]<br>B. longum subsp. longum JDM301 4/11 [69/294, 139/324, 44/300, 205/363]<br>B. longum subsp. longum BBMN68 1/1 [361/393]<br>B. longum subsp. infantis 157F 4/5 [77/270, 78/363, 221/336, 141/219]<br>B. longum BXY01 4/8 [69/294, 205/363, 139/324, 44/270]<br>B. longum subsp. infantis ATCC 15697 4/13 [69/294, 82/354, 166/363, 80/324]<br>B. pseudocatenulatum JCM 1200 2/2 [261/665, 330/330]
SRS024075		B. adolescentis 22L 1/1 [114/180]

		B. kashiwanohense JCM 15439 5/11 [241/291, 220/309, 191/270, 120/330, 28/318]<br>B. pseudocatenulatum JCM 1200 2/2 [318/665, 330/330]
SRS024132		B. kashiwanohense JCM 15439 3/11 [188/291, 28/309, 24/294]<br>B. adolescentis BBMN23 3/4 [203/309, 182/291, 300/300]
SRS024331		B. longum subsp. longum KACC 91563 1/4 [324/393]<br>B. longum subsp. longum BBMN68 1/1 [224/393]<br>B. longum subsp. longum JCM 1217 1/1 [224/393]<br>B. adolescentis BBMN23 2/4 [228/246, 291/291]<br>B. adolescentis ATCC 15703 1/1 [519/519]
SRS024625		B. pseudocatenulatum JCM 1200 2/2 [102/665, 245/330]
SRS045004		B. pseudocatenulatum JCM 1200 2/2 [190/665, 298/330]
SRS045645		B. longum subsp. longum GT15 1/1 [258/363]<br>B. longum subsp. longum BBMN68 1/1 [315/393]<br>B. longum BXY01 2/8 [238/363, 23/270]<br>B. kashiwanohense JCM 15439 5/11 [266/291, 103/309, 100/270, 78/330, 26/318]<br>B. longum subsp. longum KACC 91563 2/4 [277/393, 145/219]<br>B. pseudocatenulatum JCM 1200 2/2 [318/665, 330/330]
SRS045713		B. breve NCFB 2258 3/10 [42/294, 198/264, 100/324]<br>B. breve JCM 7017 2/9 [112/270, 212/324]<br>B. breve JCM 7019 2/7 [112/264, 225/324]<br>B. breve JCM 1192 2/7 [112/264, 186/324]<br>B. longum subsp. infantis 157F 4/5 [251/270, 185/363, 42/336, 116/219]<br>B. longum BXY01 3/8 [185/363, 309/324, 112/270]<br>B. longum subsp. longum JDM301 3/11 [309/324, 112/300, 185/363]<br>B. breve UCC2003 2/7 [112/270, 251/324]<br>B. breve ACS-071-V-Sch8b 2/9 [283/324, 112/270]<br>B. breve 12L 1/6 [309/324]<br>B. bifidum PRL2010 3/4 [51/297, 157/324, 88/375]<br>B. adolescentis BBMN23 2/4 [78/246, 222/291]

		B. longum subsp. longum KACC 91563 3/4 [33/297, 76/393, 215/219]
SRS047014	L. rhamnosus LOCK908 6/10 [116/249, 57/348, 66/219, 36/273, 75/219, 77/270]<br>L. rhamnosus LOCK900 4/12 [66/219, 36/273, 116/249, 124/255]<br>L. rhamnosus Lc 705 6/10 [116/249, 77/270, 36/273, 57/348, 124/255, 75/216]	B. adolescentis BBMN23 3/4 [122/309, 28/291, 241/300]
SRS048164		B. longum subsp. longum JDM301 2/11 [220/324, 295/363]<br>B. breve JCM 7017 1/9 [322/324]<br>B. longum BXY01 2/8 [295/363, 220/324]<br>B. longum subsp. infantis JCM 1222 2/14 [152/324, 293/363]<br>B. longum subsp. infantis ATCC 15697 2/13 [293/363, 152/324]<br>B. longum subsp. longum JCM 1217 1/1 [266/393]<br>B. longum DJO10A 1/1 [363/363]<br>B. longum NCC2705 1/1 [333/363]<br>B. longum subsp. infantis 157F 2/5 [363/363, 133/219]<br>B. longum subsp. longum GT15 1/1 [324/363]<br>B. longum subsp. longum BBMN68 1/1 [236/393]<br>B. longum subsp. longum KACC 91563 2/4 [224/393, 171/219]
SRS049164		B. longum strain BXY01 1/3 [181/285]<br>B. adolescentis BBMN23 4/4 [100/309, 60/246, 60/291, 229/300]
SRS049712		B. breve JCM 7019 2/7 [209/294, 145/324]<br>B. longum subsp. infantis ATCC 15697 4/13 [175/294, 95/363, 122/324, 97/324]<br>B. kashiwanohense JCM 15439 5/11 [122/324, 168/297, 231/324, 270/270, 24/294]<br>B. longum subsp. infantis 157F 2/5 [132/363, 206/336]<br>B. bifidum BGN4 3/4 [111/180, 284/297, 324/324]<br>B. bifidum JCM 1255 4/4 [107/360, 212/297, 324/324, 111/180]<br>B. breve UCC2003 3/7 [209/294, 122/324, 231/324]<br>B. longum BXY01 3/8 [209/294, 100/363, 231/324]<br>B. bifidum PRL2010 4/4 [230/297, 324/324, 120/375, 175/180]<br>B. longum subsp. longum JDM301 4/11

		[209/294, 231/324, 100/363, 122/324]<br>B. breve ACS-071-V-Sch8b 3/9 [75/294, 122/324, 145/324]<br>B. longum subsp. infantis JCM 1222 4/14 [175/294, 122/324, 97/324, 95/363]<br>B. bifidum ATCC 29521 4/4 [212/297, 303/303, 107/360, 111/180]<br>B. breve 689b 2/5 [145/324, 209/294]<br>B. bifidum S17 4/4 [111/180, 145/324, 122/375, 212/297]
SRS049900		B. bifidum S17 4/4 [99/180, 160/324, 195/375, 146/297]<br>B. longum subsp. longum KACC 91563 2/4 [76/393, 176/219]<br>B. bifidum BGN4 3/4 [99/180, 66/297, 205/324]<br>B. bifidum JCM 1255 4/4 [180/360, 146/297, 205/324, 99/180]<br>B. bifidum ATCC 29521 4/4 [146/297, 183/303, 180/360, 99/180]<br>B. bifidum PRL2010 4/4 [66/297, 205/324, 195/375, 135/180]
SRS049995		B. bifidum PRL2010 3/4 [156/297, 163/324, 121/180]<br>B. bifidum ATCC 29521 3/4 [219/297, 142/303, 59/180]<br>B. adolescentis ATCC 15703 1/1 [420/519]<br>B. adolescentis BBMN23 1/4 [188/291]
SRS050422		B. bifidum PRL2010 4/4 [200/297, 142/324, 312/375, 152/180]<br>B. bifidum S17 4/4 [180/180, 324/324, 321/375, 297/297]<br>B. bifidum BGN4 4/4 [180/180, 180/180, 236/297, 142/324]<br>B. bifidum JCM 1255 4/4 [341/360, 297/297, 142/324, 180/180]<br>B. bifidum ATCC 29521 4/4 [297/297, 121/303, 341/360, 180/180]<br>B. breve JCM 1192 4/7 [64/276, 54/264, 166/324, 125/294]<br>B. breve NCFB 2258 5/10 [49/171, 82/294, 80/264, 166/324, 64/276]<br>B. breve JCM 7019 5/7 [155/369, 170/294, 54/264, 61/285, 270/324]<br>B. breve 689b 3/5 [169/324, 54/264, 170/294]<br>B. longum subsp. infantis ATCC 15697 6/13 [43/294, 38/348, 249/363, 100/369, 125/324, 61/285]<br>B. longum DJO10A 1/1 [363/363]<br>B. longum subsp. longum JCM 1217 1/1 [372/393]<br>B. longum subsp. infantis 157F 5/5 [127/270, 61/285, 363/363, 147/336, 206/219]<br>B. longum subsp. longum KACC 91563

		3/4 [30/297, 393/393, 219/219]<br>B. longum subsp. longum BBMN68 1/1 [345/393]<br>B. longum subsp. longum GT15 1/1 [343/363]<br>B. longum BXY01 4/8 [170/294, 344/363, 167/324, 26/270]<br>B. longum subsp. longum JDM301 5/11 [170/294, 94/285, 167/324, 56/300, 344/363]<br>B. adolescentis BBMN23 4/4 [299/309, 229/246, 286/291, 300/300]<br>B. longum subsp. infantis JCM 1222 6/14 [43/294, 100/369, 61/285, 125/324, 249/363, 26/270]<br>B. adolescentis ATCC 15703 1/1 [389/519]
SRS050752		B. longum subsp. infantis 157F 3/5 [169/270, 27/363, 67/336]<br>B. adolescentis ATCC 15703 1/1 [511/519]<br>B. adolescentis BBMN23 4/4 [181/309, 187/246, 185/291, 299/300]
SRS050925		B. longum strain BXY01 1/3 [212/285]<br>B. longum subsp. longum JDM301 4/11 [21/294, 126/186, 212/285, 28/363]<br>B. longum subsp. infantis ATCC 15697 2/13 [122/363, 368/369]<br>B. longum subsp. infantis 157F 3/5 [225/285, 122/363, 21/336]<br>B. longum subsp. infantis JCM 1222 2/14 [368/369, 122/363]<br>B. breve JCM 7019 3/7 [369/369, 21/294, 38/285]<br>B. longum BXY01 3/8 [21/294, 28/363, 126/186]
SRS051031		B. pseudocatenulatum JCM 1200 2/2 [318/665, 330/330]<br>B. adolescentis ATCC 15703 1/1 [412/519]
SRS052027		B. pseudocatenulatum JCM 1200 2/2 [114/665, 236/330]
SRS052697		B. longum subsp. longum JDM301 3/11 [21/294, 25/300, 280/363]<br>B. longum subsp. infantis 157F 4/5 [25/270, 304/363, 21/336, 217/219]<br>B. longum DJO10A 1/1 [304/363]<br>B. longum NCC2705 1/1 [304/363]<br>B. longum BXY01 3/8 [21/294, 280/363, 25/270]<br>B. adolescentis 22L 1/1 [112/180]<br>B. longum subsp. infantis JCM 1222 2/14 [251/363, 25/270]<br>B. longum subsp. infantis ATCC 15697 2/13 [25/264, 251/363]<br>B. kashiwanohense JCM 15439 6/11 [241/291, 240/309, 209/270, 24/294, 204/330, 47/318]

		B. longum subsp. longum GT15 1/1 [305/363]<br>B. longum subsp. longum KACC 91563 2/4 [365/393, 219/219]<br>B. longum subsp. longum BBMN68 1/1 [379/393]<br>B. longum subsp. longum JCM 1217 1/1 [357/393]<br>B. longum 105-A 1/1 [272/363]<br>B. pseudocatenulatum JCM 1200 2/2 [318/665, 330/330]
SRS053214		B. bifidum PRL2010 3/4 [324/324, 178/375, 66/180]<br>B. bifidum ATCC 29521 3/4 [303/303, 178/360, 66/180]<br>B. breve NCFB 2258 3/10 [21/294, 178/264, 93/324]<br>B. bifidum BGN4 3/4 [66/180, 70/180, 324/324]<br>B. bifidum JCM 1255 3/4 [178/360, 324/324, 66/180]<br>B. longum subsp. infantis 157F 4/5 [235/270, 153/363, 195/336, 75/219]<br>B. adolescentis BBMN23 2/4 [184/246, 279/291]
SRS053398		B. kashiwanohense JCM 15439 2/11 [195/324, 24/294]<br>B. bifidum BGN4 2/4 [102/180, 291/324]<br>B. bifidum JCM 1255 2/4 [24/360, 291/324]<br>B. bifidum PRL2010 2/4 [291/324, 24/375]<br>B. bifidum ATCC 29521 2/4 [270/303, 24/360]<br>B. breve 689b 2/5 [129/324, 21/294]<br>B. angulatum JCM 7096 1/2 [206/318]
SRS056519		B. longum subsp. longum KACC 91563 2/4 [125/393, 167/219]<br>B. longum subsp. infantis 157F 2/5 [342/363, 167/219]<br>B. longum DJO10A 1/1 [342/363]<br>B. longum subsp. longum JCM 1217 1/1 [287/393]<br>B. longum subsp. longum GT15 1/1 [196/363]<br>B. longum 105-A 1/1 [301/363]<br>B. adolescentis BBMN23 1/4 [247/291]
SRS057717		B. pseudocatenulatum JCM 1200 2/2 [162/665, 216/330]
SRS058723		B. bifidum S17 1/4 [22/180]<br>B. adolescentis BBMN23 4/4 [309/309, 246/246, 274/291, 300/300]
SRS058770		B. longum strain BXY01 1/3 [188/285]<br>B. longum BXY01 3/8 [133/294, 303/363, 112/186]<br>B. longum subsp. longum JDM301 4/11 [133/294, 112/186, 188/285, 303/363]

		B. longum subsp. infantis 157F 5/5 [172/270, 181/285, 308/363, 209/336, 156/219]<br>B. longum subsp. infantis JCM 1222 4/14 [133/294, 101/369, 69/285, 252/363]<br>B. longum DJO10A 1/1 [308/363]<br>B. longum subsp. longum JCM 1217 1/1 [383/393]<br>B. longum NCC2705 1/1 [308/363]<br>B. longum subsp. longum KACC 91563 2/4 [313/393, 219/219]<br>B. longum subsp. infantis ATCC 15697 5/13 [133/294, 38/348, 252/363, 101/369, 69/285]<br>B. longum subsp. longum GT15 1/1 [216/363]<br>B. longum subsp. longum BBMN68 1/1 [383/393]
SRS063040		B. adolescentis BBMN23 4/4 [56/309, 117/246, 182/291, 193/300]
SRS064276		B. adolescentis BBMN23 4/4 [309/309, 230/246, 264/291, 234/300]<br>B. longum subsp. longum KACC 91563 2/4 [51/393, 219/219]<br>B. breve NCFB 2258 4/10 [21/294, 197/264, 141/324, 86/276]<br>B. longum subsp. infantis 157F 4/5 [235/270, 79/363, 195/336, 198/219]<br>B. breve JCM 7019 3/7 [121/294, 100/264, 285/324]<br>B. longum BXY01 4/8 [121/294, 79/363, 164/324, 100/270]<br>B. bifidum PRL2010 4/4 [150/297, 83/324, 292/375, 180/180]<br>B. bifidum S17 4/4 [144/180, 236/324, 292/375, 172/297]<br>B. bifidum BGN4 4/4 [144/180, 30/180, 269/297, 83/324]<br>B. bifidum JCM 1255 4/4 [277/360, 172/297, 83/324, 144/180]<br>B. bifidum ATCC 29521 4/4 [172/297, 62/303, 277/360, 144/180]<br>B. adolescentis ATCC 15703 1/1 [467/519]
SRS075398		B. pseudocatenulatum JCM 1200 2/2 [318/665, 311/330]<br>B. kashiwanohense JCM 15439 5/11 [22/318, 172/297, 270/270, 55/330, 34/318]
SRS077730		B. longum subsp. infantis 157F 4/5 [22/270, 248/285, 154/363, 107/219]<br>B. longum subsp. longum KACC 91563 2/4 [45/393, 183/219]<br>B. breve JCM 7019 2/7 [289/369, 93/285]<br>B. longum subsp. infantis JCM 1222 3/14 [266/369, 93/285, 22/270]

		B. longum subsp. infantis ATCC 15697 3/13 [22/264, 266/369, 93/285]
SRS078176		B. adolescentis ATCC 15703 1/1 [459/519]<br>B. adolescentis BBMN23 2/4 [246/246, 249/291]<br>B. longum subsp. longum KACC 91563 1/4 [183/219]

1. [n1]/[n0] n1 - number of detected genetic markers; n0 - number of all genetic markers

2. coverage of genetic marker number i
