# Supplementary material for: Employing toxin-antitoxin genome markers for identification of Bifidobacterium and Lactobacillus strains in human metagenomes
Source: PeerJ. 2019 Mar 4;7:e6554. doi: 10.7717/peerj.6554 (PMC6404652; doi:10.7717/peerj.6554)
Supplement: Supplemental Information 4 — Representation of TASs type II genes of superfamilies RelBE and MazEF in Lactobacillus strains (A) and Bifidobacterium strains (B). Black boxes show the presence of a gene. The name of each group of TA genes consists of the name of a gene and the number of a group. [file peerj-07-6554-s004.pdf]

A

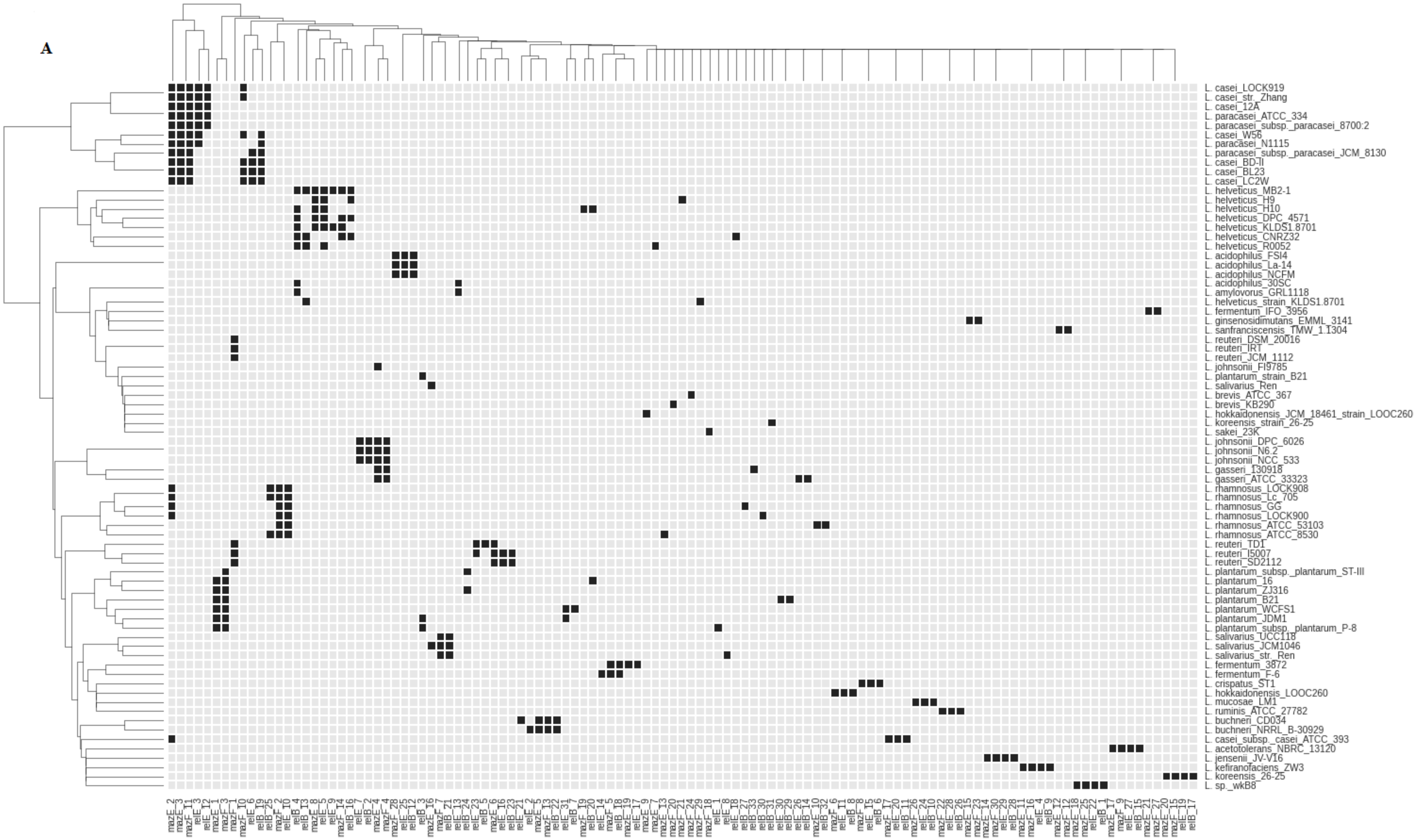
L. casei_LOCK919
L. casei_str._Zhang
L. casei_12A
L. paracasei_ATCC_334
L. paracasei_subsp._paracasei_8700:2
L. casei_W56
L. paracasei_N1115
L. paracasei_subsp._paracasei_JCM_8130
L. casei_BD-II
L. casei_BL23
L. casei_LC2W
L. helveticus_MB2-1
L. helveticus_H9
L. helveticus_H10
L. helveticus_DPC_4571
L. helveticus_KLDS1.8701
L. helveticus_CNRZ32
L. helveticus_R0052
L. acidophilus_FSI4
L. acidophilus_La-14
L. acidophilus_NCFM
L. acidophilus_30SC
L. amylovorus_GRL1118
L. helveticus_strain_KLDS1.8701
L. fermentum_IFO_3956
L. ginsenosidimutans_EMML_3141
L. sanfranciscensis_TMW_1.1304
L. reuteri_DSM_20016
L. reuteri_IRT
L. reuteri_JCM_1112
L. johnsonii_FI9785
L. plantarum_strain_B21
L. salivarius_Ren
L. brevis_ATCC_367
L. brevis_KB290
L. hokkaidonensis_JCM_18461_strain_LOOC260
L. koreensis_strain_26-25
L. sakei_23K
L. johnsonii_DPC_6026
L. johnsonii_N6.2
L. johnsonii_NCC_533
L. gasseri_130918
L. gasseri_ATCC_33323
L. rhamnosus_LOCK908
L. rhamnosus_Lc_705
L. rhamnosus_GG
L. rhamnosus_LOCK900
L. rhamnosus_ATCC_53103
L. rhamnosus_ATCC_8530
L. reuteri_TD1
L. reuteri_I5007
L. reuteri_SD2112
L. plantarum_subsp._plantarum_ST-III
L. plantarum_16
L. plantarum_ZJ316
L. plantarum_B21
L. plantarum_WCFS1
L. plantarum_JDM1
L. plantarum_subsp._plantarum_P-8
L. salivarius_UCC118
L. salivarius_JCM1046
L. salivarius_str._Ren
L. fermentum_3872
L. fermentum_F-6
L. crispatus_ST1
L. hokkaidonensis_LOOC260
L. mucosae_LM1
L. ruminis_ATCC_27782
L. buchneri_CD034
L. buchneri_NRRL_B-30929
L. casei_subsp._casei_ATCC_393
L. acetotolerans_NBRC_13120
L. jensenii_JV-V16
L. kefiranofaciens_ZW3
L. koreensis_26-25
L. sp._wkB8

B

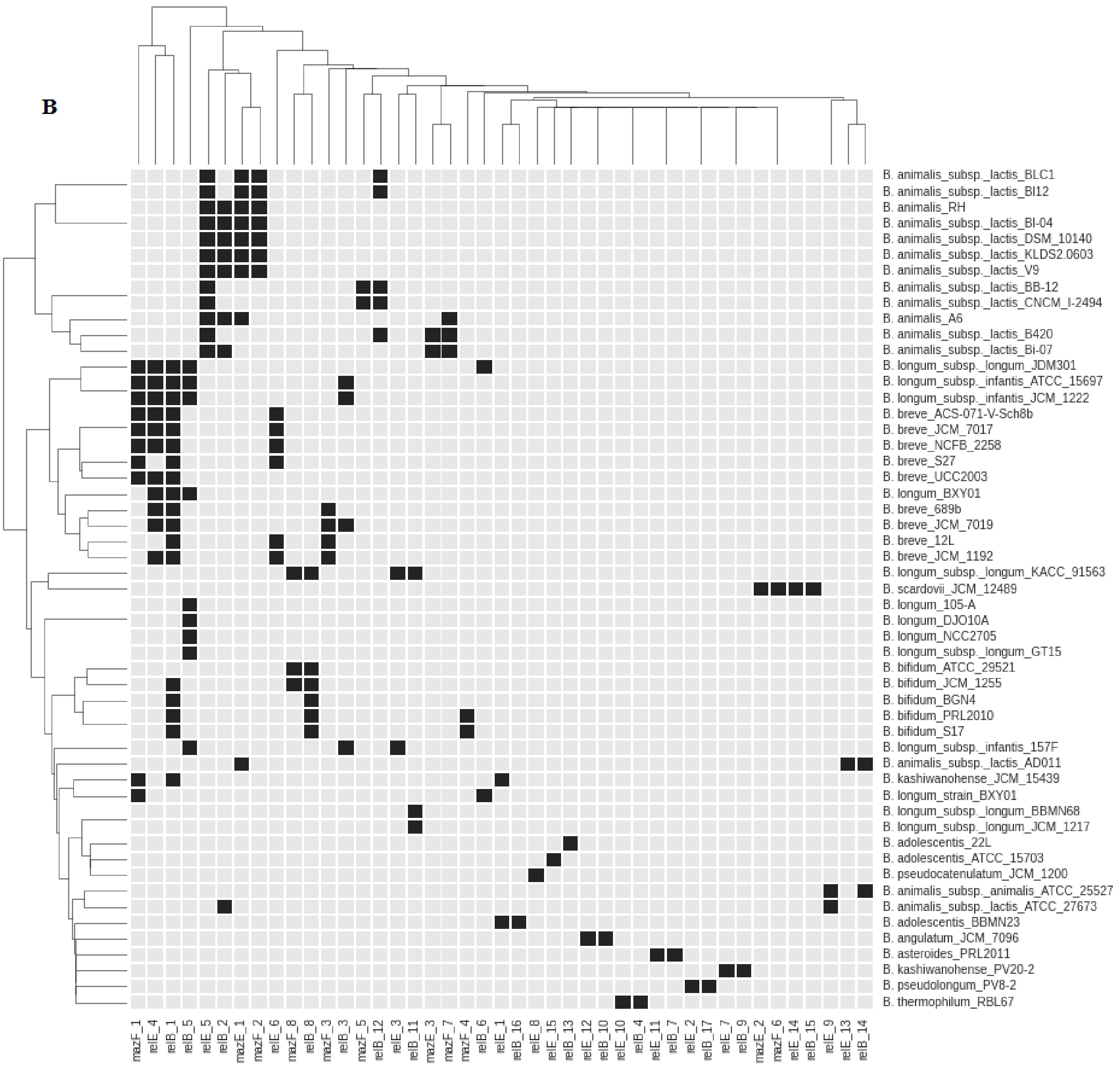

Figure S1 Representation of TASs type II genes of superfamilies RelBE and MazEF in *Lactobacillus* strains **(A)** and *Bifidobacterium* strains **(B)**. Black boxes show the presence of a gene. The name of each group of TA genes consists of the name of a gene and the number of a group.
